# Chronic Pulmonary Aspergillosis in Children: A Scoping Global Review

**DOI:** 10.1093/ofid/ofag186

**Published:** 2026-04-06

**Authors:** Filip Pavlovic, Richard Kwizera, David W Denning

**Affiliations:** School of Medicine, The University of Manchester, Manchester, UK; Infectious Diseases Institute, College of Health Sciences, Makerere University, P.O. Box 22418, Kampala, Uganda; Manchester Fungal Infection Group, Faculty of Biology, Medicine and Health, The University of Manchester, Manchester Academic Health Science Centre, Manchester, UK

**Keywords:** aspergilloma, *Aspergillus fumigatus*, hyper-IgE syndrome, lobectomy, mortality

## Abstract

**Background:**

Chronic pulmonary aspergillosis (CPA) affects those with underlying lung conditions or mild immunocompromise. Chronic pulmonary aspergillosis carries a poor prognosis in adults. Its pathogenesis remains obscure. We summarize all cases of CPA in children globally.

**Methods:**

From 6503 screened reports, we reviewed the full text of 604, of which 44 fit the inclusion criteria and an additional 11 other cases with little detail which were included under Supplementary Data. Chronic granulomatous disease and cystic fibrosis cases were excluded.

**Results:**

We found 47 well-documented individual cases of CPA in children published from 1963 to 2022. Twenty-two cases were simple aspergillomas, and 11 were chronic cavitary pulmonary aspergillosis. Ages ranged from under 1 year to 17 years old, and 28 (59.8%) were male. Eighteen (38.3%) cases had no reported underlying disease. Underlying diseases included pulmonary tuberculosis (14.9%), Job's Syndrome (10.6%), congenital pulmonary airway malformation (8.5%), allergic bronchopulmonary aspergillosis or asthma (6.4%), pulmonary hydatid cyst (4.3%), bacterial pneumonia with cavitation (4.3%), diabetes mellitus (4.3%), and single cases of pulmonary sequestration or bronchogenic cyst. All cases had either microbiological or immunological evidence of *Aspergillus* spp. apart from 2 confirmed by histopathology only. Surgical resection only was done in 18 (39.1%) patients, 15 (32.6%) were treated with surgery and antifungal therapy, and 13 (28.3%) were only treated with antifungals; one patient died before intervention. Forty-three cases (91.5%) were alive on hospital discharge, but follow-up was limited, while 2 died.

**Conclusions:**

Chronic pulmonary aspergillosis is apparently rare in children but does occur, often with no antecedent condition. Prognosis is good with early diagnosis.

## Teaser

Chronic pulmonary aspergillosis occurs occasionally in children, at any age, often with no antecedent condition, or linked to tuberculosis or Job's Syndrome. Most patients have a simple aspergilloma or chronic cavitary phenotype. Surgical resection is often favored; immediate prognosis is good, but follow-up data are rare.

Chronic pulmonary aspergillosis (CPA) is a type of long-term lung fungal infection caused by *Aspergillus* spp. [1]. *Aspergillus fumigatus* is the predominant CPA pathogen with *Aspergillus flavus* and *Aspergillus niger* more frequent in hotter climates. Chronic pulmonary aspergillosis is seen in immunocompetent adults, often with underlying lung conditions such as chronic obstructive pulmonary disease (COPD), tuberculosis (TB), non-tuberculous mycobacterial infections, sarcoidosis after pneumothorax, and other lung diseases, all rare conditions in children as causes of lung cavitation [2].

The global burden of CPA was estimated at 1.84 million people worldwide as of 2024, with annual crude estimates of deaths being 340,000 per year [3]. The majority of CPA cases originate from TB-affected countries [4, 5]. The prognosis of CPA is poor; mortality rate is estimated at 22% overall, 14%–15% at 1 year, 35%–38% at 5 years, and 53% at 10 years [6]. Age is also an important factor in mortality, with a 10% increase in mortality each decade, and only a 1% 5-year mortality in those <40 years of age [6].

Chronic pulmonary aspergillosis in children is rarely reported, as many of the risk factors linked to CPA in adults, especially cavitary TB and COPD, are rare in children. Given the limited published literature on CPA in children, we aimed to review and summarize the existing evidence on the burden, presentation, underlying conditions, diagnosis, treatment options, and clinical outcomes of CPA among children aged 0–17 years globally.

## METHODS

### Study Design and Inclusion and Exclusion Criteria

This was a qualitative scoping review performed according to the PRISMA checklist. We aimed to include all studies of any design describing CPA in children (0–17 years) following the diagnostic criteria, highlighting information such as clinical symptoms, underlying disease, imaging findings, method of laboratory diagnosis, type of CPA, treatment options, and outcomes. There was no restriction on the year of publication and language used. The diagnosis of CPA conformed to that provided by Denning et al [7], including subacute invasive, simple aspergilloma, and *Aspergillus* nodules.

We applied the following exclusion criteria: patient age 18 years or over; cause of the infection was not confirmed to be *Aspergillus*c spp.; patient was a transplant recipient; patient had an ongoing oncological condition with treatments that caused immunosuppression; cases involving iatrogenic immunosuppression (including sustained, high dose corticosteroids); invasive pulmonary aspergillosis, including those involving chronic granulomatous disease; cases of cystic fibrosis; signs of dissemination beyond the lungs indicating invasive aspergillosis; cases of allergic aspergillosis without radiological features of CPA; all reviews and reports without new case details; and reports with insufficient detail about the case to analyze (these were noted separately). The main reason for the exclusion criteria was to limit cases of invasive pulmonary aspergillosis, as these make up a large amount of the literature. This is why cancer and transplant cases were rejected, as well as cases of CGD. Cystic fibrosis was excluded due to the higher likelihood of an alternative diagnosis of allergic aspergillosis which can be difficult to distinguish from CPA in those with very abnromal lungs. Cases involving iatrogenic immunosuppression were also rejected due to it being a well-known cause of secondary infections, including aspergillosis.

### Search Strategy

To capture as many relevant citations as possible, PubMed, Scopus, OVID Medline, and Embase electronic searches were executed on the 2nd of February 2025 to identify all studies addressing CPA in children globally. In PubMed, we used the following search strategies: ((Aspergilloma) OR (Aspergillus) OR (Aspergillosis) OR (Aspergillus AND Mycetoma)) NOT (“Invasive Aspergillosis”) NOT (“Allergic Pulmonary Aspergillosis”) NOT (“Allergic Bronchopulmonary Aspergillosis”).

The search strings used in Scopus, OVID Medline, and Embase are detailed in the Supplementary Data.

### Review of Studies

All the studies obtained through the literature search were imported into citation software “EndNote.” The initial screening of publications was via their title and abstract, allowing exclusion of many reports. The full text of those remaining was studied for their eligibility. Any additional cases found among the references were examined and added if relevant. Any non-English studies were either translated by native speakers; otherwise, they were processed through Google Translate.

Two reviewers (F. P. and R. K.) screened articles without blinding to capture relevant studies. Disagreements between the 2 review authors were resolved by the involvement of a third author (D. W. D.) where necessary. The database was then screened again using full text for each study to include only relevant articles.

### Data Summary and Analysis

Data from the final studies were summarized in an Excel spreadsheet, recording all pertinent clinical and microbiologic data provided by the authors. Data were analyzed qualitatively to answer the objectives.

## RESULTS

**Figure 1.**
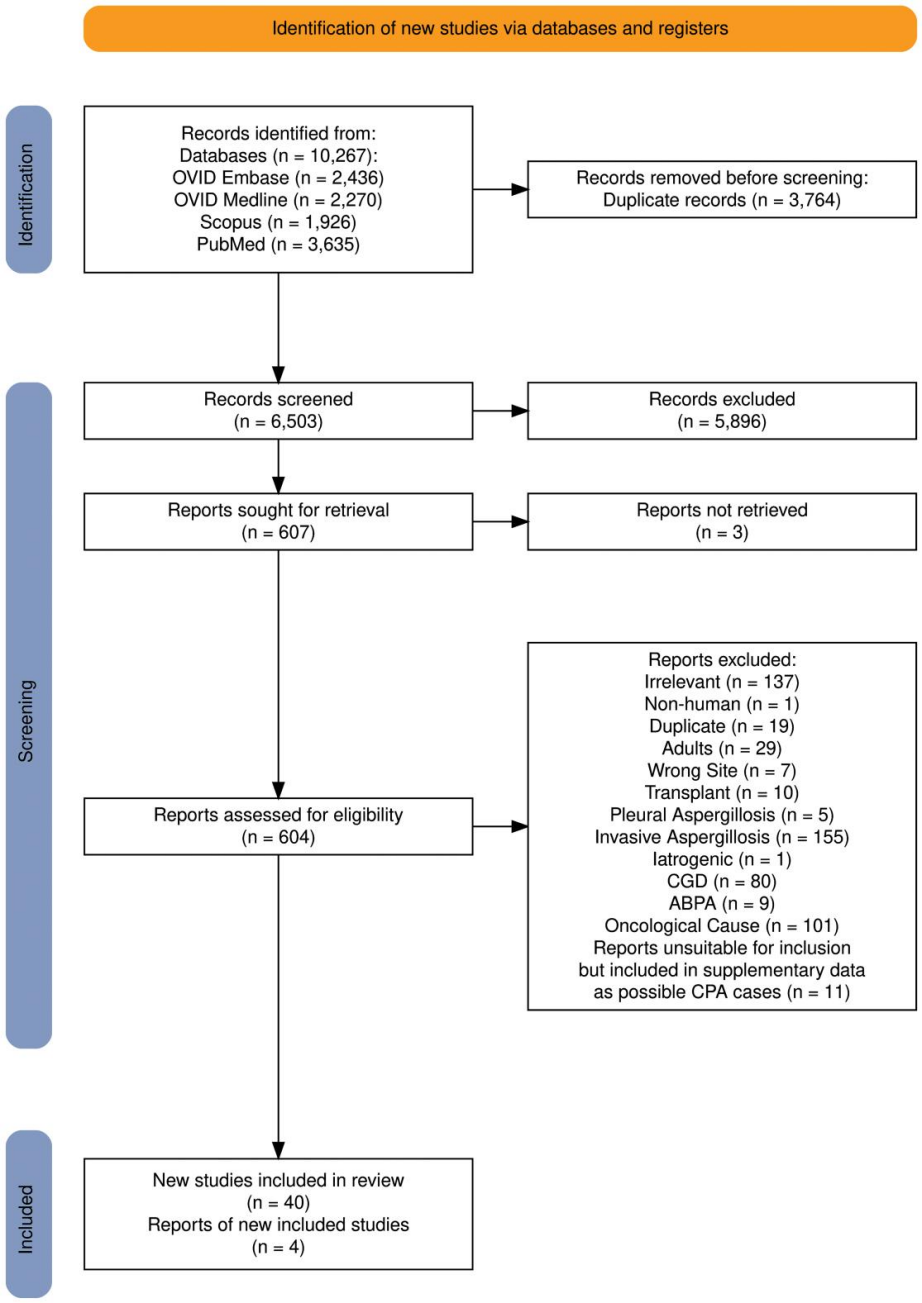
PRISMA flow diagram, showing citation selection process for the review.

Our literature search produced 10 267 potential studies from 4 different databases, of which 3764 studies were duplicates. This left 6503 studies for screening via title and abstract, of which we eliminated 5896 of 6503 studies as out of scope. We examined 607 studies, but 3 were unavailable despite extensive efforts. This left 604 studies that were eligible for assessment using our inclusion and exclusion criteria. Our criteria excluded 552 studies, leaving 51 studies plus an additional 4 studies found by examining the reference listings. Following the case definition provided by Denning et al [7] (Supplementary Table 1), 11 of these studies did not contain enough information to determine whether they were CPA cases or not and so were excluded in the final articles but were included in “possible CPA cases” (Supplementary Table 2) (Figure 1).

**Figure 2.**
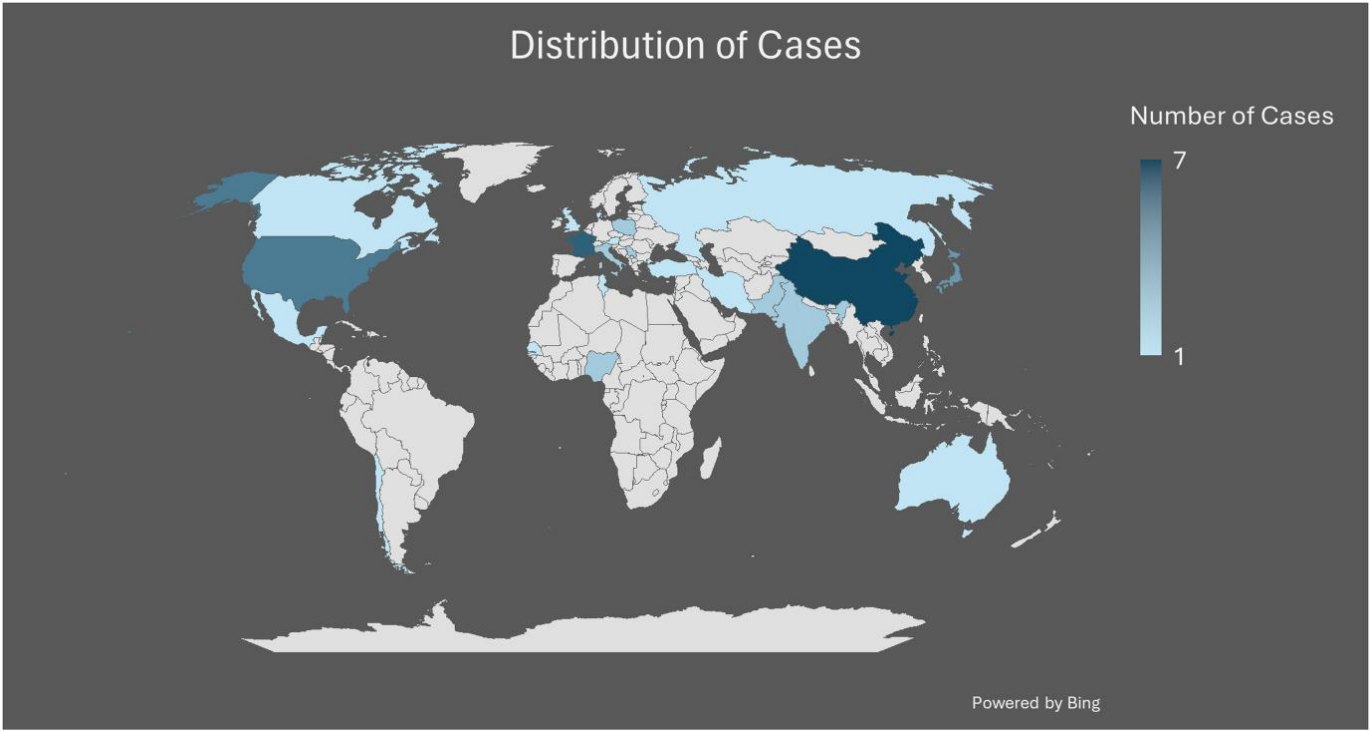
Distribution of cases.

We identified 44 case reports and case series. These came from 23 countries with the highest number of cases reported from France (n = 6), China (n = 7), the USA (n = 5), and Japan (n = 4). Italy, Poland, Serbia, Pakistan, India, and Nigeria had 2 cases each. The rest had one case each (Figure 2).

The first case was published in 1963 [8] and the last 3 cases in 2022. This resulted in 47 individual cases across 44 case reports/series (Table 1).

**Table 1.** Case Table Summarizing Information Obtained From Found Cases

Reference	Year of Presenting	Age in Years	Sex	Country	Presenting Symptoms	Past Medical History/Underlying Disease	Imaging Findings	Microbiology Findings	Immunology Findings	Type of CPA	Treatment	Case Outcome
Chaptal et al (1963) [8]	1962	2	F	France	Cough with hemoptysis	Acute bronchitis	Aspergilloma	*A fumigatus* isolated in gastric secretions	N/D	Simple aspergilloma	Prednisolone and hexacycline. Lobectomy of the right lower lobe	Alive
Miyamoto et al (1963) [9]	1960	13	M	Japan	Fever and productive cough	Pulmonary TB	Aspergilloma	Pathology suggestive of *Aspergillus*	N/D	Simple aspergilloma	Left upper lobe lobectomy	Alive
Krenmayr (1964) [10]	1961	10	M	Austria	Loss of appetite, weight loss, night sweats, pyrexia	Pertussis, measles, and myocarditis. Tuberculosis in 1959	Aspergilloma	*Aspergillus* spp. on histology	Unremarkable	Simple aspergilloma	Resection of the left anterior medial-basal segment	Alive
Besa et al (1966) [11]	1962	4	F	Italy	1st presentation—spiking temperatures, dry cough, and shortness of breath 2nd presentation—productive cough with hemoptysis, respiratory failure	N/D	1st presentation—right-sided lung abscess 2nd presentation—aspergilloma	*Pseudomonas piocianneus* and *A fumigatus*	Unremarkable	Simple aspergilloma	Right upper lobe lobectomy	Alive
Fouquet et al (1966) [12]	1962	11	F	France	N/D	Pulmonary TB	Multiple nodules	*A fumigatus* isolated from pus	Unremarkable	*Aspergillus* nodules	Surgical resection via left lateral thoracotomy	Alive
Rzepecki et al (1967) [13]	1966	11	M	Poland	Hemoptysis, fever, productive cough	N/D	Thin-walled cavity containing an aspergilloma in right lung	Negative precipitins test. Microscopy of resected material showed *Aspergillus*	Unremarkable	Simple aspergilloma	Middle lobectomy of the right lung	Alive
Isvanski et al (1970) [14]	1965	9	F	Serbia	Cough, diabetic coma	Diabetes mellitus, chickenpox, measles	Consolidation on the right, a spot on the left apex. Cavity in the upper left lung and right base lung	Histological finding of *Aspergillus* in autopsy (figures provided)	N/D	Subacute invasive pulmonary aspergillosis	Dead before treatment could be started	Died 12 d after admission
Evans et al (1971) [15]	1970	8	M	UK	N/D	Skin infections due to *Staphylococcus aureus*. Tension cysts from staphylococcus in both lungs—destroyed the right upper lobe	Aspergilloma	*A fumigatus* isolated from sputum	Unremarkable	Simple aspergilloma	Clotrimazole	Alive
Berger et al (1972) [16]	1968–1969	12	F	USA	Fever in 1968 and hemoptysis in 1969	Initially diagnosis of asthma—changed to ABPA	Multiple nodules—left upper lobe in 1968 and single nodule formed into fibrotic scar—right upper lobe in 1968–1969	*A fumigatus* was isolated from the abscesses in the left upper lobe. Smears and bronchial washings grew *A fumigatus* in right upper lobe during the single nodule. After resection of the fibrotic scar *A niger* and *Staphylococcus aureus* was grown	Unremarkable	*Aspergillus* nodules leading to CFPA	Amphotericin B, potassium iodide, and resection of the affected lobes	Alive
Beauvais et al (1975) [17]	1974	10	F	France	Fever, asthenia, and hemoptysis	N/D	Aspergilloma	All negative. Histological studies pointed toward *Aspergillus*	Unremarkable	Simple aspergilloma	Resection of the affected right upper lobe	Alive
Kane et al (1976) [18]	1973	15	F	Senegal	Weight loss and fever for 4 m	Diabetes mellitus	Aspergilloma	Negative cultures, positive *Aspergillus* antigen	N/D	Simple aspergilloma	Anti-TB medication. Left upper lobectomy was performed	Alive
Rzepecki (1977) [19]	N/D	16	M	Poland	N/D	N/D	Non-specific round, homogenous sharp edge	Transthoracic biopsy grew *Aspergillus*	N/D	Simple aspergilloma	Enucleation of the “cyst” from 3rd segment of the left lung	Alive
Bundgaard et al (1983) [20]	N/D	16	F	Denmark	Productive, worsening cough, chest pain	Nonrelevant	Lung abscess	Aspirated pus grew *A fumigatus*	N/D	CCPA	Thoracotomy. Amphotericin B along with 5-fluorocytosine and prednisone	Alive
Grignet et al (1983) [21]	1979	16	F	France	Flu-like symptoms	Pulmonary sequestration	Right basal cavity.	Positive *Aspergillus* precipitins	N/D	Simple aspergilloma	Aerosolized amphotericin B and operation to fix the pulmonary sequestration	Alive
David et al (1986) [22]	1981	10	M	France	Presented due to an automobile accident, so asymptomatic	N/D	Large thin-walled cavity between right middle and upper lobe containing 2 aspergillomas	Strongly positive *Aspergillus* precipitins	N/D	Simple aspergilloma	Resection of both cavities removing the material	Alive
Castaneda-Ramos et al (1989) [23]	N/D	12–15	M	Mexico	Hemoptysis	Asthma	Cavity in the left upper lobe.	Culture grew *A fumigatus* Negative precipitins + high total IgE	Unremarkable	Simple aspergilloma	Amphotericin B followed by upper left lobectomy	Alive
Liu et al (1990) [24]	1989	11	M	Canada	Routine check-up, probably none.	Job's Syndrome	Large cavity in right lower lobe with aspergilloma	Pathological review of the right lower lobe confirmed *Aspergillus*	Elevated IgE serum levels	Simple aspergilloma	Right lower lobectomy	Not mentioned
Hiura et al (1993) [25]	1986	17	M	Japan	Left-sided chest pain	N/D	Left-sided pneumothorax on X-ray. CT showed a cystic shadow in the left apex	Histological study revealed *Aspergillus*	Unremarkable	*Aspergillus* nodules	Cystectomy was performed to remove the cyst. 5-Flucytosine was used after surgery	Alive Pne umothorax persisted
Karim et al (1997) [26]	N/D	8	F	Pakistan	Cough and fever for 7 m. Dyspnea for 2 m	Pulmonary TB	Old tuberculosis lesions, a bulla on the right side and left side	Right side grew *A fumigatus* and *Streptococcus pneumoniae*	N/D	Subacute invasive aspergillosis	Right side thoracotomy with bullectomy	Alive
Santambrogio et al (1997) [27]	1995	11	F	Italy	Productive cough and hemoptysis	Job's Syndrome	Right lower lobe pneumatocele	Sputum grew *A fumigatus*	N/D	CCPA	Right lower lobectomy	Alive
Wolach et al (1998) [28]	N/D	15	M	Israel	2-d cough, chest pain, and dyspnea	Job's Syndrome	X-ray—right pneumothorax with atelectasis CT—right pneumothorax and large cavity containing aspergilloma	Tissue culture grew *A fumigatus*	N/D	Simple aspergilloma	Right upper lobectomy	Not mentioned
Kul’ko et al (2003) [29]	N/D	17	M	Russia	Weakness, fever, and hemoptysis	N/D	X-ray -> 7 cm non-homogeneous round formation in the upper lobe of the left lung	Sputum and bronchoalveolar lavage (BAL) grew *A terreus* BAL grew *A restrictus* Resected material grew *A terreus*	N/D	Simple aspergilloma	Amphotericin B followed by left upper lobectomy	Alive
Zhao et al (2005) [30]	N/D	3	M	China	High fever and mild cough	Pulmonary TB	Right upper lobe—pleural thickening, cavitation, and multiple nodular shadows	Sputum and lung tissue cultures grew *A fumigatus*	Unremarkable.	*Aspergillus* nodules	Amphotericin B and itraconazole	Alive Symptoms were controlled 10 –30 d after treatment
Rubilar et al (2006) [31]	2002	8	F	Chile	Fever, cough, shortness of breath, bronchorrhea with foul-smelling mold	Laryngeal papillomatosis, pneumonia caused by influenza A, pseudomembranous tracheitis due to *S. aureus*	Chest X-ray—round annular shape in right upper lobe CT showed cavitary images in the left lung and a subpleural nodule	Sputum culture provided with *A fumigatus*	Unremarkable	*Aspergillus* nodules	Itraconazole	Alive
Arai et al (2007) [32]	2006	6	F	Japan	Fever and cough	Recurrent bouts of pneumonia, previous surgery for pulmonary cyst in left upper lobe. Previous right-sided pneumothorax. Job's Syndrome	A cavity in the right lung field and another in the left with clear mass inside	*A fumigatus* was grown from the sputum culture	Elevated serum IL-6 and hyper-IgE levels	Aspergilloma	Amikacin, meropenem, micafungin, voriconazole, and amphotericin B. Right middle lobectomy was performed, and a left upper lobectomy was performed	Alive Gene ral condition improved, but cavities remained
Chtourou et al (2007) [33]	N/D	17	M	Tunisia	Productive cough, with fever and right-sided chest pain	Persistent pulmonary TB	Pyopneumothorax, right apical cavitary lesion. Bronchopleural fistula	Drainage from the pyopneumothorax revealed *A niger*	N/D	Subacute invasive aspergillosis	Anti-TB medication with right upper lobectomy	Alive
Kuruvilla et al (2008) [34]	N/D	13	M	India	Massive hemoptysis, breathless on exertion, and hoarseness of voice	HPV infection of the vocal cord since age of 3	Cavity containing an aspergilloma. Nodules around the cavity	Histological study revealed *Aspergillus*	N/D	Subacute invasive aspergillosis	Surgical resection—left lower lobectomy followed by itraconazole	Alive
Yonker et al (2011) [35]	N/D	14	M	USA	Fever, cough, and throat pain	N/D	Aspergilloma and cystic cavity in right upper lobe	Bronchoalveolar lavage grew *A fumigatus*. Histology revealed aspergilloma and CPAM	Unremarkable	Simple aspergilloma	Itraconazole, right upper lobectomy	Alive
Pan et al (2013) [36]	N/D	15	M	China	Cough, hemoptysis, and slight fever for 1 m	N/D	Solitary pulmonary mass with cavitation in right lower lobe	Negative sputum tests, ELISA test was weakly positive for *echinococcal* antibodies. Histology revealed *Aspergillus*	N/D	CCPA	Surgical resection of the right lower lobe followed by albendazole	Alive
Kane et al (2014) [37]	N/D	15	F	USA	14 m of chronic cough	N/D	Two cavitary lesion in the left lower lobe. MRI revealed an CPAM	Sputum grew *A versicolor*	Unremarkable	CCPA	Voriconazole and a lobectomy	Alive Resolved symptoms
Stojnic et al (2014) [38]	N/D	10	M	Serbia	Cough and hemoptysis	N/D	Round lesion in apical and posterior segments of the right upper lung	Cultures of BAL were negative. *Aspergillus* IgM was positive, and histology was diagnostic. The lung tissue sample culture grew *A fumigatus*	Unremarkable	Subacute invasive aspergillosis	Voriconazole	Alive
Emiralioglu et al (2017) [39]	N/D	14	F	Turkey	Shortness of breath and hemoptysis	Previous hydatid cyst in upper lobe of both lungs. Cavitary pneumonia treated with antibiotic	Chest X-ray revealed mass like opacity in the upper and middle lobe of right lung. CT revealed large mass with necrotic features in right upper and middle lobe	Biopsy revealed *Aspergillus*. Positive *Aspergillus* antibodies	Unremarkable	Subacute invasive aspergillosis	Voriconazole	Alive De creased size of mass
Chandrakar et al (2018) [40]	N/D	12	M	India	Shortness of breath, fever, and cough	N/D	Well-defined irregular, marginated, non-enhancing lesion. Suggestive of a hydatid cyst	Histological study revealed *Aspergillus* within the hydatid cyst	N/D	*Aspergillus* nodule	Left lower lobectomy	Not mentioned
Isnard et al (2018) [41]	N/D	15	N/D	France	Recurrent hemoptysis	Previous chest wound at age 11	Residual pneumatocele colonized by *Aspergillus*	Galactomannan bronchoalveolar lavage fluid—2436 (normal valve: 0.5–1) Histological evidence of *Aspergillus*	N/D	Simple aspergilloma	Azole antifungals were not tolerated; therefore, a right lower lobectomy was undertaken followed by liposomal amphotericin B	Alive
McDowell et al, 2018 [42]	N/D	16	M	USA	Cough and hemoptysis	None	Right upper lobe—showed a cavitation—possible aspergilloma	IgG, Culture and histology revealed *A fumigatus.*	N/D	Simple aspergilloma	Resection of upper right lobe.	Alive
Yu et al (2019) [43]	N/D	9	M	USA	Cough, weight loss, and fever	ABPA	Right upper lobe—large cavity. Smaller lesion appeared on CT	Cultures positive for *A fumigatus*	High IgE—characteristic of Job's Syndrome	Subacute invasive aspergillosis	Micafungin and voriconazole	Alive
Aboksari et al (2020) [44]	N/D	10	M	Iran	Chest pain, fever, cough, and malaise for 3 m	Hydatid cyst	Air-containing cyst in right lower lobe on X-ray	Histological study revealed *Aspergillus* within the hydatid cyst	Unremarkable.	CCPA	Total cystectomy was conducted	Alive Symptoms resolved
Rana et al (2020) [45]	N/D	10	M	Pakistan	Fever, shortness of breath, and cough for 1 m	Job's Syndrome	CT scan—large pneumatocele	Culture revealed *A nidulans*. Fungal lung abscess was revealed	N/D	Simple aspergilloma	Left lung decortication—followed by voriconazole	Alive
Abo et al (2021) [46]	N/D	0.1	M	Australia	Rapid breathing and labored breathing from birth	None	X-ray revealed cystic lesion in the right lower lobe. CT revealed a 30 mm thick-walled cavity	PCR from the resection of the right lower lobe revealed *Aspergillus*	Unremarkable	CCPA	Right lower lobectomy followed by voriconazole—voriconazole was stopped due to photosensitive side effect	Alive
Liu et al (2021) [47]	N/D	0.58	F	China	Cough for 3 m, with fever and shortness of breath for 4 d	N/D	X-ray revealed bilateral inflammation and local consolidation. CT revealed a large thick-walled cavity in the right lower lobe	*Aspergillus* IgG was detected	N/D	CCPA	Oral voriconazole	Alive Clinical and radiologic condition improved
Zhang et al (2021) [48]	N/D	10	F	China	Cough and hemoptysis	Recurrent pneumonia and allergic sinusitis	Aspergilloma	BAL culture negative Histopathology confirmed CPAM *Aspergillus* IgE and IgG positive	2nd presentation possible ABPA	Simple aspergilloma	Voriconazole for 8 wks After relapse—voriconazole, itraconazole, and prednisone	Alive
Zhang et al (2021) [48]	N/D	13	F	China	Cough and hemoptysis	Allergic sinusitis and skin allergy	Aspergilloma nodules	Surgical pus culture positive for *A fumigatus* Histopathology confirmed CPAM *Aspergillus* IgE positive	Unremarkable	Simple aspergilloma and *Aspergillus* nodule(s)	Voriconazole for 8 wks After relapse Voriconazole and itraconazole for 6 m	Alive
Zhang et al (2021) [48]	N/D	9	M	China	Cough and chest pain	Recurrent pneumonia, allergic sinusitis, and skin allergy	Cavitary lesion	Histopathology confirmed CPAM *Aspergillus* IgG positive	Unremarkable	CCPA	Voriconazole for 12 wks	Alive
Zhang et al (2021) [48]	N/D	13	M	China	Signs of pneumothorax	None	Pneumothorax	Histopathology confirmed *Aspergillus* *Aspergillus* IgG positive	Unremarkable	N/D	Voriconazole for 8 wks	Alive
Adeyemo et al (2022) [49]	2020	13	F	Nigeria	Fever and cough	Pulmonary TB	Cavitary lesions within both upper lung zones	*A flavus* was grown on culture	N/D	CCPA	Intravenous voriconazole	Died 23 d after admission
Ando et al (2022) [50]	2018	16	M	Japan	Chest pain and shortness of breath	ABPA, asthma	Chest X-ray showed a cavity with consolidation in the right upper lung field. CT revealed a cavity with a fungal ball. 3 d later, more consolidation appeared around the cavity	*A fumigatus* was grown from the sputum and BALF culture	Unremarkable	CCPA	Voriconazole with prednisone followed by the partial resection of the right upper lobe	Alive
Davies et al (2022) [51]	N/D	9	M	Nigeria	Recurrent cough, abdominal pain, and fever. Chest pain, night sweats	None	multiple thick-walled pulmonary cavitary nodules in both lungs	*Aspergillus* IgG was detected	N/D	CCPA	Oral itraconazole	Alive Improvement within 48 h

Abbreviations: CPAM, congenital pulmonary airway malformation; TB, tuberculosis; CFPA, chronic fibrosis pulmonary aspergillosis; CCPA, chronic cavitary pulmonary aspergillosis; CT, computed tomography; IgE, immunoglobulin E; BAL, bronchoalveolar lavage; IL-6, interleukin-6; HPV, human papillomavirus; ELISA, enzyme-linked immunosorbent assay; ABPA, allergic bronchopulmonary aspergillosis; IgG, immunoglobulin G.

### CPA Characteristics in Children

**Figure 3.**
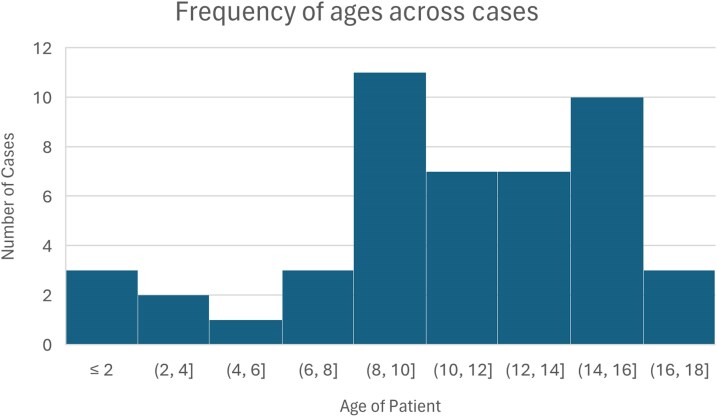
Histogram detailing the age distribution of cases.

Of the 47 cases of CPA in children, 22 (46.8%) had simple aspergillomas, 11 (23.4%) CCPA, 6 (12.8%) SAIA, 5 (10.6%) *Aspergillus* nodules and one (2.1%) CFPA. In 2 cases (4.2%), the type of CPA could not be determined. Twenty-eight of the cases (59.8%) were male and 18 (38.3%) were female, with one without data. The age range varied between 0.1-to-17 years old (Figure 3).

### Underlying Conditions

The past medical history of the patients varied. Nineteen (40.4%) had no relevant previous medical history or no mention of one. Seven patients (14.9%) had a background of TB, 5 (10.6%) Job's Syndrome (hyper-IgE syndrome), 4 (8.5%) were confirmed to have CPAM, 3 (6.4%) had concurrent ABPA or asthma, 2 (4.3%) a history of a hydatid cyst, 2 (4.3%) prior cavitary pneumonia such as *Pseudomonas* or *Staphylococcus aureus* pneumonia, 2 (4.3%) diabetes mellitus, 1 (2.1%) pulmonary sequestration, and another (2.1%) a bronchogenic cyst. A small number of patients had other past medical histories irrelevant to the development of CPA. Among all of the cases, including 11 possible CPA cases, the most common initial differential diagnosis was TB [52–62].

### Clinical Presentation

The most common clinical presentations of CPA in children were cough (n = 27), fever (n = 23), hemoptysis (n = 16), shortness of breath (n = 11), and chest pain (n = 9). Two patients presented for other reasons and were asymptomatic.

### Diagnosis

All 47 cases provided evidence of imaging consistent with the presence of CPA and subacute invasive aspergillosis: one or more cavities (n = 22), an aspergilloma (n = 18), nodules (n = 7), consolidation (n = 3), and pleural thickening (n = 1).

In addition, all cases provided either microbiological or immunological evidence of *Aspergillus* spp., except 2 cases, which did not mention any evidence (4.2%) but had typical radiological and clinical features. The different diagnostic modalities included culture, histology, *Aspergillus* PCR, *Aspergillus* antigen, and *Aspergillus* antibody. Six CPA cases were diagnosed using *Aspergillus* IgG testing, and an additional 2 cases were diagnosed using the precipitins test, which detects different types of *Aspergillus* antibodies, including IgG. *Aspergillus* IgM was also used in one case, while *Aspergillus* IgE was used in 3 cases. One case was diagnosed using *Aspergillus* serology which was not specified. The causative agents identified by culture included *A fumigatus* (n = 20), unidentified *Aspergillus* species (n = 17), *A niger* complex (n = 2), *A versicolor* (n = 2), *A nidulans* (n = 2), *A flavus* (n = 2), *A terreus* (n = 1), and *A restrictus* (n = 1).

### Treatment

One patient died before treatment could be given. We identified 18 (39.1%) patients who were treated with surgical intervention, including lobectomy and cystectomy. Fifteen (32.6%) patients were treated with combined surgery and antifungal therapy. The last 13 (28.3%) patients were treated with antifungal therapy only. The most common antifungal drugs used were voriconazole (n = 13), itraconazole (n = 8), amphotericin B (n = 8), flucytosine (n = 2), micafungin (n = 2), and clotrimazole (n = 1).

### Clinical Outcomes

Among all cases identified, 43/47 (91.5%) were alive and improved following treatment, but follow-up was limited. Two (4.3%) cases died, while 3 (6.4%) had unknown outcomes.

## DISCUSSION

Our broad search of the literature identified 47 cases of CPA in children, with at least another 11 possible cases. There are most likely older cases of CPA in children that have been mislabeled as invasive aspergillosis due to the differing understanding and unclear case definitions of aspergillosis clinical phenotypes in past decades. As in adults, some children were misdiagnosed with TB and given anti-TB medication [33]. It is likely that CPA cases are underreported or misdiagnosed due to the low index of clinical suspicion and limited knowledge about the disease. Therefore, it is possible that the reported number of cases in this scoping review underestimates the true prevalence of pediatric CPA, likely due to misclassifications of invasive aspergillosis, unclear case definitions, and misdiagnoses of TB.

*Aspergillus*-specific IgG antibody testing is a vital and valuable tool in the laboratory diagnosis of CPA. Although 8 cases (16.7%) were diagnosed using *Aspergillus* IgG or precipitins testing, the cutoff values for *Aspergillus* IgG antibody testing for CPA in children have not been established. Those used in the identified studies varied from 0.8 to 27 mgA/L depending on the kit used [47, 51, 63]. However, *Aspergillus* IgG antibody testing has been used in the diagnosis of ABPA among children with asthma as described in a few cross-sectional and retrospective studies [64–67]. Even in the pediatric ABPA population, the cutoffs are not standardized. A cutoff value of 16.8 mgA/L on ImmunoCAp was shown to have a sensitivity of 63.6% and specificity of 68.5% for ABPA [64]. Another pediatric study using ELISA showed a cutoff value of <8 U/mL, negative; 8–12 U/mL, equivocal; and >12 U/mL, positive [65]. EIA has been shown to have a cutoff value of 15.7 AU/mL in children with ABPA.

Tuberculosis is known to be the major risk factor for CPA. Tuberculosis was the commonest underlying diagnosis in the identified studies but affects children differently than adults. Children are much more likely than adults to develop active TB after being exposed to *Mycobacterium tuberculosis*, the likelihood being 40%–50% if less than 12 months old, dropping gradually to 10%–15% in adolescence [68]. If the direct cause of CPA within children was usually TB, the age distribution of CPA cases with a history of TB might reflect this skewed age distribution; however, this is not the case for the CPA cases. Notably, some children developed CPA within 0.8–2 years of being diagnosed with primary TB [9, 12, 49]. Children are less likely to develop cavities as a complication of TB, as cavitation tends to increase with age in adulthood [69]. Cavitation substantially increases the risk of subsequent CPA in adults [70]. The percentage of children with TB that present with cavitation on imaging has been placed between 5% and 21% [69, 71, 72], with possibly some regional factors implicated. MTB–CPA co-infection is common in adults because cavitary post-primary TB provides a substrate for *Aspergillus* infection, whereas it is rare in children due to non-cavitary primary TB, shorter disease duration, and under-recognition. The largest problem with the diagnosis of CPA in all ages is to differentiate between TB and aspergillosis [73]. Children with cavitary lesions following TB should be regularly reviewed to identify CPA early, as recommended in adults. A recent study of the sequelae of pulmonary TB in 73 children (aged 6–16 years) in Kenya found abnormal chest radiographs in 21% with fibrosis and pleural thickening the most common findings, but not cavitation [74]. This discrepancy arises because cavitary TB in children is rare and highly age-dependent, and reported prevalence varies with inclusion of adolescents, imaging sensitivity, and classification practices, rather than true inconsistency in disease biology.

Pulmonary hydatid cysts infection usually occurs in childhood, but only about 10%–20% of cases are diagnosed under 16 years old [75]. Co-infection with *Aspergillus* leading to CPA is rare and mostly documented in adults (78 cases) [76]. Chronic pulmonary aspergillosis also complicated cavitary pneumonia in children in 5 cases. The most common causes of cavitary pneumonia are *Streptococcus pneumoniae*, *S aureus*, and *Klebsiella pneumoniae* [77], often linked with prior influenza.

Job's Syndrome, or hyper-IgE syndrome, is an immunodeficiency disorder characterized by high IgE levels caused in 2/3 seconds of cases by genetic variants in the STAT3 gene and a birth incidence of ∼1 per million [78]. The long-term pulmonary complications are numerous; the most significant are recurrent infection, abscess, bronchiectasis, and pneumatoceles, which are at risk of being infected with *Aspergillus* spp. We identified 5 cases of CPA in childhood linked to Job's Syndrome which should be considered as an additional diagnosis if CPA presents in children. The patients’ reported past medical histories may be incomplete, as the underlying disease linked to CPA was often discovered after CPA presented, as in cases linked to Job's Syndrome.

Congenital pulmonary airway malformation (CPAM) is malformations that occur during the development of the lungs. They lead to areas of cystic or adenomatous areas in the lungs [79]. We identified a case series of 4 children with CPA [48] complicating CPAMs.

Bronchogenic cysts were also linked to CPA in 1 child [67] and are different from CPAM as they result from abnormal budding of the ventral foregut that forms the bronchial tree.

One CPA case [9] was linked to pulmonary sequestration, sometimes referred to as an accessory (portion of) lung, and refers to extra-segmentary lung tissue. This lung tissue can be intralobar (within the lung) or extralobar (outside of the lung) [80]. Symptoms vary from cough and recurrent pneumonia to dyspnea, cyanosis, and feeding difficulties. These can mask CPA and lead to misdiagnosis.

We found 19 published cases of CPA in children with no obvious cause mentioned or discovered after surgery. One explanation might be that this is an idiopathic infection, as documented in <5% of adults with CPA who have no apparent underlying cause [2, 81]. The higher proportion of idiopathic CPA in children compared with adults is generally not because children truly have more “cause-free” disease, but because the usual adult predisposing factors are less common, harder to identify, or behave differently in pediatric patients. Some genetic susceptibility markers for CPA have been identified [82–84]). A partial genetic cause remains a possible explanation for some cases of CPA in children.

It is difficult to comment on the effectiveness of the different medical treatments or surgical options used (including lobectomy, antifungal therapy, cystectomy, combination therapy) and long-term outcomes of CPA in children with little to no follow-up in most cases, a significant limitation in the data. However, the short-term mortality rate appears to be low, with only 2 (4.2%) cases leading to death among the 47 cases collected. Most of the studies only mention a single follow-up visit with the patients; the longest follow-up was 8 years. This makes it difficult to establish an estimate of relapse, mortality, or prognosis, as there may be long-term consequences of CPA in children not yet documented.

Published data are limited globally about CPA in children. Most of the available literature is published in case reports/series with limited epidemiological studies. Only 2 cases came from Africa and one from South America, yet these are the tropical regions where the burden of TB (the major risk factor for CPA in adults) is very high. Therefore, the burden of CPA in children in these areas is probably underestimated. Evidence is lacking on how to clinically and radiologically distinguish CPA from TB in a resource-limited setting with limited access to microbiology and radiology services. There is no discrete case definition for CPA in children; most cases are diagnosed based on the adult case definition. The treatment of CPA in children is not standardized.

## CONCLUSIONS

These 47 individual published cases of confirmed CPA in children demonstrate that there are enough cases in the literature to support raising awareness of this condition among pediatricians. We have also found unique causes that are not found in adults, such as congenital malformations of the lungs (CPAM, bronchogenic cysts, and pulmonary sequestration) and a relatively high frequency in Job's Syndrome. The cases reported do not give any insight into the patients’ quality of life after treatment or whether the patients were still alive years later.

## Supplementary Material

ofag186_Supplementary_Data

## Supplementary Data

Supplementary materials are available at *Open Forum Infectious Diseases* online. Consisting of data provided by the authors to benefit the reader, the posted materials are not copyedited and are the sole responsibility of the authors, so questions or comments should be addressed to the corresponding authors.
