# Supplementary material for: Chronic Pulmonary Aspergillosis in Children: A Scoping Global Review
Source: Open Forum Infect Dis. 2026 Apr 6;13(5):ofag186. doi: 10.1093/ofid/ofag186 (PMC13138880; doi:10.1093/ofid/ofag186)
Supplement: ofag186_Supplementary_Data [file ofag186_supplementary_data.docx]

Table of Contents

[Detailed methods 1](#_Toc203943079)

[Supplementary Table 1: Cases of CPA with references included 4](#_Toc203943080)

[Supplementary Table 2: Other Possible CPA cases found in literature 14](#_Toc203943081)

[References 15](#_Toc203943082)

# **Detailed methods**

In order to conduct the literature search, four databases were selected to identify cases of CPA in children. The databases are PubMed, Scopus, OVID Medline and Embase. For each database, different searching strategies were implemented in order to account for the variability in the designs of the search engines. Each of the following searches were conducted on the 2^nd^ of February 2025.

For PubMed: ((Aspergilloma) OR (Aspergillus) OR (Aspergillosis) OR (Aspergillus AND Mycetoma)) NOT ("Invasive Aspergillosis") NOT ("Allergic Pulmonary Aspergillosis") NOT ("Allergic Bronchopulmonary Aspergillosis") was constructed. The following filters were applied:

- Remove Preprints
- Child Only (Birth – 18yrs)
- Humans Only

This was done to ensure the widest possible search regarding aspergillosis but removing conditions that are not considered relevant. This is to ensure that we do not miss out on any potential cases via our search methods.

For Scopus: TITLE-ABS-KEY ( aspergilloma OR aspergillosis OR ( aspergillus AND mycetoma ) ) AND ( EXCLUDE ( EXACTKEYWORD , "Aged, 80 And Over" ) OR EXCLUDE ( EXACTKEYWORD , "Adult" ) OR EXCLUDE ( EXACTKEYWORD , "Nonhuman" ) OR EXCLUDE ( EXACTKEYWORD , "Animal" ) OR LIMIT-TO ( EXACTKEYWORD , "Child" ) OR LIMIT-TO ( EXACTKEYWORD , "School Child" ) OR LIMIT-TO ( EXACTKEYWORD , "Infant" ) OR LIMIT-TO ( EXACTKEYWORD , "Adolescent" ) ) was constructed. Similar filters were applied for this database as with the previous database.

For OVID Medline, the search method was constructed via a series of search commands that were combined with each to achieve a desired search result. A series of thirteen commands were created which achieved the following search method:

1 Aspergillosis/

2 Pulmonary Aspergillosis/

3 Aspergillus/

4 Mycetoma/

5 3 and 4

6 limit 1 to ("all infant (birth to 23 months)" or "all child (0 to 18 years)")

7 limit 2 to ("all infant (birth to 23 months)" or "all child (0 to 18 years)")

8 limit 3 to ("all infant (birth to 23 months)" or "all child (0 to 18 years)")

9 limit 4 to ("all infant (birth to 23 months)" or "all child (0 to 18 years)")

10 limit 5 to ("all infant (birth to 23 months)" or "all child (0 to 18 years)")

11 6 or 7 or 10

12 exp animals/ not humans.sh.

13 11 not 12

For OVID Embase, the search method was constructed in a similar way as for OVID Medline:

1 aspergillosis/

2 chronic pulmonary aspergillosis/

3 lung aspergillosis/

4 chronic cavitary pulmonary aspergillosis/

5 chronic necrotizing pulmonary aspergillosis/

6 bronchopulmonary aspergillosis/

7 chronic progressive pulmonary aspergillosis/

8 aspergilloma/

9 Aspergillus/

10 mycetoma/

11 9 and 10

12 limit 1 to (infant <to one year> or preschool child <1 to 6 years> or school child <7 to 12 years> or adolescent <13 to 17 years>)

13 limit 2 to (infant <to one year> or preschool child <1 to 6 years> or school child <7 to 12 years> or adolescent <13 to 17 years>)

14 limit 3 to (infant <to one year> or preschool child <1 to 6 years> or school child <7 to 12 years> or adolescent <13 to 17 years>)

15 limit 4 to (infant <to one year> or preschool child <1 to 6 years> or school child <7 to 12 years> or adolescent <13 to 17 years>)

16 limit 5 to (infant <to one year> or preschool child <1 to 6 years> or school child <7 to 12 years> or adolescent <13 to 17 years>)

17 limit 6 to (infant <to one year> or preschool child <1 to 6 years> or school child <7 to 12 years> or adolescent <13 to 17 years>)

18 limit 7 to (infant <to one year> or preschool child <1 to 6 years> or school child <7 to 12 years> or adolescent <13 to 17 years>)

19 limit 8 to (infant <to one year> or preschool child <1 to 6 years> or school child <7 to 12 years> or adolescent <13 to 17 years>)

20 limit 9 to (infant <to one year> or preschool child <1 to 6 years> or school child <7 to 12 years> or adolescent <13 to 17 years>)

21 limit 10 to (infant <to one year> or preschool child <1 to 6 years> or school child <7 to 12 years> or adolescent <13 to 17 years>)

22 limit 11 to (infant <to one year> or preschool child <1 to 6 years> or school child <7 to 12 years> or adolescent <13 to 17 years>)

23 12 or 13 or 14 or 15 or 16 or 17 or 18 or 19 or 22

24 (exp animal/ or exp invertebrate/ or nonhuman/ or animal experiment/ or animal tissue/ or animal model/ or exp plant/) not (exp human/ or human tissue/)

25 23 not 24

# **Supplementary Table 1**: Cases of CPA with references included

| **Reference** | **Year of Presenting** | **Age in years** | **Sex** | **Country** | **Presenting Symptoms** | **Past Medical History/ underlying disease** | **Imaging Findings** | **Microbiology Findings** | **Immunology Findings** | **Type of CPA** | **Treatment** | **Case Outcome** |
| --- | --- | --- | --- | --- | --- | --- | --- | --- | --- | --- | --- | --- |
| Chaptal et al, 1963 (1) | 1962 | 2 | F | France | Cough with haemoptysis | Acute Bronchitis | Aspergilloma | *A. fumigatus* isolated in gastric secretions | N/D | Simple aspergilloma | Prednisolone and hexacycline**.** Lobectomy of the right lower lobe. | Alive |
| Miyamoto et al, 1963 (2) | 1960 | 13 | M | Japan | Fever and productive cough | Pulmonary TB | Aspergilloma | Not mentioned | N/D | Simple aspergilloma | Left Upper Lobe lobectomy. | Alive |
| Krenmayr 1964 (3) | 1961 | 10 | M | Austria | Loss of appetite, weight loss, night sweats, pyrexia | Pertussis, Measles and Myocarditis. Tuberculosis in 1959 | Aspergilloma | *Aspergillus spp.* on histology | Unremarkable | Simple aspergilloma | Resection of the left anterior medial-basal segment. | Alive |
| Besa et al, 1966 (4) | 1962 | 4 | F | Italy | 1st presentation - spiking temperatures, dry cough and shortness of breath.  2nd presentation - productive cough with haemoptysis, respiratory failure | N/D | 1st presentation - Right sided lung abscess.  2nd presentation - aspergilloma | *Pseudomonas piocianneus* and *A. fumigatus.* | Unremarkable | Simple aspergilloma | Right upper lobe lobectomy. | Alive |
| Fouquet et al, 1966 (5) | 1962 | 11 | F | France | N/D | Pulmonary TB | Multiple nodules. | *A. fumigatus* isolated from pus | Unremarkable | *Aspergillus* nodules | Surgical resection via left lateral thoracotomy. | Alive |
| Rzepecki et al, 1967 (6) | 1966 | 11 | M | Poland | Haemoptysis, fever, productive cough. | N/D | Thin-walled cavity containing an aspergilloma | Negative precipitins test | Unremarkable | Simple aspergilloma | Middle lobectomy of the right lung. | Alive |
| Isvanski et al, 1970 (7) | 1965 | 9 | F | Serbia | Cough, diabetic coma | Diabetes Mellitus, chickenpox, measles | Consolidation on the right, a spot on the left apex. Cavity in the upper left lung and right base lung. | N/D | N/D | Subacute invasive pulmonary aspergillosis | Dead before treatment could be started | Died 12 days after admission |
| Evans et al, 1971 (8) | 1970 | 8 | M | UK | N/D | Skin infections due to Staphylococcus aureus. Tension cysts from staphylococcus in both lungs - destroyed the right upper lobe. | Aspergilloma | *A. fumigatus* isolated from sputum | Unremarkable | Simple aspergilloma | Clotrimazole | Alive |
| Berger et al, 1972 (9) | 1968-1969 | 12 | F | USA | Fever in 1968 and haemoptysis in 1969 | Initially diagnosis of asthma – changed to ABPA | Multiple nodules - left upper lobe in 1968 and single nodule formed into fibrotic scar - right upper lobe in 1968 - 1969 | *A. fumigatus* was isolated from the abscesses in the left upper lobe. Smears and bronchial washings grew *A. fumigatus* in right upper lobe during the single nodule. After resection of the fibrotic scar *A. niger* and *Staphylococcus aureus* was grown. | Unremarkable | *Aspergillus* nodules leading to CFPA | Amphotericin B, Potassium Iodide and resection of the affected lobes. | Alive |
| Beauvais et al, 1975 (10) | 1974 | 10 | F | France | Fever, asthenia and haemoptysis | N/D | Aspergilloma | All negative. Histological studies pointed towards *Aspergillus*. | Unremarkable | Simple aspergilloma | Resection of the affected right upper lobe. | Alive |
| Kane et al, 1976 (11) | 1973 | 15 | F | Senegal | Weight loss and fever for 4 months | Diabetes Mellitus | Aspergilloma | Negative cultures, positive *Aspergillus* antigen | N/D | Simple aspergilloma | Anti-TB Medication. Left upper lobectomy was performed. | Alive |
| Rzepecki 1977 (12) | N/D | 16 | M | Poland | N/D | N/D | Non-specific Round, homogenous sharp edge. | Transthoracic biopsy grew *Aspergillus*. | N/D | Simple aspergilloma | Enucleation of the "cyst" from 3rd segment of the left lung. | Alive |
| Bundgaard et al, 1983 (13) | N/D | 16 | F | Denmark | Productive, worsening cough, chest pain. | Nonrelevant | Lung abscess | Aspirated pus grew *A. fumigatus* | N/D | CCPA | Thoracotomy. Amphotericin B along with 5-fluorocytosine and prednisone. | Alive |
| Grignet et al, 1983 (14) | 1979 | 16 | F | France | Flu-like symptoms | Pulmonary sequestration | Right basal cavity. | Positive *Aspergillus* precipitins | N/D | Simple aspergilloma | Aerolised amphotericin B and operation to fix the pulmonary sequestration | Alive |
| David et al, 1986 (15) | 1981 | 10 | M | France | Presented due to an automobile accident, so asymptomatic | N/D | Large thin-walled cavity between right middle and upper lobe containing two aspergillomas | Strongly positive *Aspergillus* precipitins | N/D | Simple aspergilloma | Resection of both cavities removing the material. | Alive |
| Castaneda-Ramos et al, 1989 (16) | N/D | 12 - 15 | M | Mexico | Haemoptysis | Asthma | Cavity in the left upper lobe. | Culture grew *A. fumigatus*  Negative precipitins + High total IgE | Unremarkable | Simple aspergilloma | Amphotericin B followed by upper left lobectomy. | Alive |
| Liu et al, 1990 (17) | 1989 | 11 | M | Canada | Routine check-up, probably none. | Job's Syndrome | Large cavity in right lower lobe with aspergilloma | N/D | Elevated IgE serum levels | Simple aspergilloma | Right lower lobectomy. | Not mentioned |
| Hiura et al, 1993 (18) | 1986 | 17 | M | Japan | Left sided chest pain | N/D | Left sided pneumothorax on X-Ray. CT showed a cystic shadow in the left apex. | Histological study revealed *Aspergillus*. | Unremarkable | *Aspergillus* nodules | Cystectomy was performed to remove the cyst. 5-flucytosine was used after surgery. | Alive  Pneumothorax persisted. |
| Karim et al, 1997 (19) | N/D | 8 | F | Pakistan | Cough and fever for 7 months. Dyspnea for 2 months | Pulmonary TB | Old tuberculosis lesions, a bulla on the right side and left side. | Right side grew *A. fumigatus* and *Streptococcus pneumoniae*. | N/D | Subacute invasive aspergillosis | Right side thoracotomy with bullectomy. | Alive |
| Santambrogio et al, 1997 (20) | 1995 | 11 | F | Italy | Productive cough and haemoptysis | Job's Syndrome | right lower lobe pneumatocoele | Sputum grew *A. fumigatus* | N/D | CCPA | Right lower lobectomy | Alive |
| Wolach et al, 1998 (21) | N/D | 15 | M | Israel | 2-day cough, chest pain and dyspnoea | Job's Syndrome | X-ray - right pneumothorax with atelectasis.  CT - right pneumothorax and large cavity containing aspergilloma | Tissue culture grew *A. fumigatus* | N/D | Simple aspergilloma | Right upper lobectomy | Not mentioned |
| Kul’ko et al, 2003 (22) | N/D | 17 | M | Russia | Weakness, fever and haemoptysis | N/D |  | Sputum and Bronchoalveolar lavage (BAL) grew *A. terreus*  BAL grew *A. restrictus*  Resected material grew *A. terreus* | N/D | Simple aspergilloma | Amphotericin B followed by left upper lobectomy. | Alive |
| Zhao et al, 2005 (23) | N/D | 3 | M | China | High fever and mild cough. | Pulmonary TB | Right upper lobe - pleural thickening, cavitation and multiple nodular shadows. | Sputum and lung tissue cultures grew *A. fumigatus* | Unremarkable. | *Aspergillus* nodules | Amphotericin B and Itraconazole | Alive  symptoms were controlled 10-30 days after treatment |
| Rubilar et al, 2006 (24) | 2002 | 8 | F | Chile | Fever, cough, shortness of breath, bronchorrhea with foul-smelling mould. | Laryngeal papillomatosis, pneumonia caused by Influenza A, pseudomembranous tracheitis due to *S. aureus* | Chest X-Ray - Round annular shape in right upper lobe.  CT showed cavitary images in the left lung and a subpleural nodule. | Sputum culture provided with *A. fumigatus.* | Unremarkable. | *Aspergillus* nodules | itraconazole | Alive |
| Arai et al, 2007 (25) | 2006 | 6 | F | Japan | Fever and cough | Recurrent bouts of pneumonia, previous surgery for pulmonary cyst in left upper lobe. Previous right sided pneumothorax. Job's Syndrome | A cavity in the right lung field and another in the left with clear mass inside. | *A. fumigatus* was grown from the sputum culture. | Elevated serum IL-6 and hyper IgE levels. | Aspergilloma | Amikacin, meropenem, micafungin, voriconazole and amphotericin B. Right middle lobectomy was performed, and a left upper lobectomy were performed. | Alive  general condition improved, but cavities remained |
| Chtourou et al, 2007 (26) | N N/D | 17 | M | Tunisia | Productive cough, with fever and right sided chest pain. | Persistent pulmonary TB | Pyopneumothorax, right apical cavitary lesion. Bronchopleural fistula | Drainage from the pyopneumothorax revealed *A. niger* | N/D | Subacute invasive aspergillosis | Anti-TB Medication with right upper lobectomy | Alive |
| Kuruvilla et al, 2008 (27) | N/D | 13 | M | India | Massive haemoptysis, breathless on exertion and hoarseness of voice. | HPV Infection of the vocal cord since age of 3 | Cavity containing an aspergillomaNodules around the cavity. | Histological study revealed *Aspergillus*. | N/D | Subacute invasive aspergillosis | Surgical resection - left lower lobectomy followed by itraconazole. | Alive |
| Yonker et al, 2011 (28) | N/D | 14 | M | USA | Fever, cough and throat pain | N/D | aspergilloma and cystic cavity in right upper lobe. | Bronchoalveolar lavage grew *A. fumigatus*. Histology revealed aspergilloma and CPAM | Unremarkable | Simple aspergilloma | Itraconazole. right upper lobectomy. | Alive |
| Pan et al, 2013 (29) | N/D | 15 | M | China | Cough, haemoptysis and slight fever for one month | N/D | Solitary pulmonary mass with cavitation | Negative sputum tests, ELISA test was weakly positive for *Echinococcal* antibodies. | N/D | CCPA | Surgical resection of the right lower lobe followed by albendazole. | Alive |
| Kane et al, 2014 (30) | N/D | 15 | F | USA | 14 months of chronic cough. | N/D | Two cavitary lesion in the left lower lobe. MRI revealed an CPAM | Sputum grew *A. versicolor*. | Unremarkable | CCPA | Voriconazole and a lobectomy | Alive  Resolved symptoms |
| Stojnic et al, 2014 (31) | N/D | 10 | M | Serbia | Cough and haemoptysis | N/D | Round lesion in apical and posterior segments of the right upper lung. | Cultures of BAL were negative. *Aspergillus* IgM was positive, and histology was diagnostic. The lung tissue sample culture grew *A. fumigatus*. | Unremarkable | Subacute invasive aspergillosis | Voriconazole | Alive |
| Emiralioglu et al, 2017 (32) | N/D | 14 | F | Turkey | Shortness of breath and haemoptysis. | Previous hydatid cyst in upper lobe of both lungs. Cavitary pneumonia treated with antibiotic. | Chest XRAY revealed mass like opacity in the upper and middle lobe of right lung. CT revealed large mass with necrotic features in right upper and middle lobe. | Biopsy revealed *Aspergillus*. Positive *Aspergillus* antibodies. | Unremarkable | Subacute invasive aspergillosis | voriconazole | Alive  decreased size of mass |
| Chandrakar et al, 2018 (33) | N/D | 12 | M | India | Shortness of breath, fever and cough | N/D | Well-defined irregular, marginated, non-enhancing lesion. Suggestive of a hydatid cyst | Histological study revealed *Aspergillus* within the hydatid cyst | N/D | *Aspergillus* nodule | Left lower lobectomy | Not mentioned |
| Isnard et al, 2018 (34) | N/D | 15 | N/D | France | Recurrent haemoptysis | Previous chest wound at age 11 | Residual pneumatocele colonised by *Aspergillus* | N/D | N/D | Simple aspergilloma | Azole antifungals were not tolerated; therefore, a right lower lobectomy was undertaken followed by liposomal amphotericin B. | Alive |
| McDowell et al, 2018 (35) | N/D | 16 | M | USA | Cough and haemoptysis | None | Right upper lobe - showed a cavitation - possible aspergilloma | IgG, Culture and histology revealed *A. fumigatus.* | N/D | Simple aspergilloma | Resection of upper right lobe. | Alive |
| Yu et al, 2019 (36) | N/D | 9 | M | USA | Cough, weight loss and fever | ABPA | Right upper lobe – large cavity. Smaller lesion appeared on CT | Cultures positive for *A. fumigatus.* | High IgE – characteristic of Job’s Syndrome | Subacute invasive aspergillosis | Micafungin and voriconazole | Alive |
| Aboksari et al, 2020 (37) | N/D | 10 | M | Iran | Chest pain, fever, cough and malaise for 3 months | Hydatid cyst | Air-containing cyst in right lower lobe on X-ray | Histological study revealed *Aspergillus* within the hydatid cyst | unremarkable. | CCPA | Total cystectomy was conducted. | Alive  symptoms resolved |
| Rana et al, 2020 (38) | N/D | 10 | M | Pakistan | Fever, shortness of breath and cough for one month | Job's Syndrome | CT scan - large pneumatocele | Culture revealed *A. nidulans*. Fungal lung abscess was revealed | Not mentioned | Simple Aspergilloma | Left lung decortication – followed by voriconazole | Alive |
| Abo et al, 2021 (39) | N/D | 0.1 | M | Australia | Rapid breathing and laboured breathing from birth. | None | X-ray revealed cystic lesion in the right lower lobe. CT revealed a 30mm thick-walled cavity. | PCR from the resection of the right lower lobe revealed *Aspergillus*. | Unremarkable | CCPA | Right lower lobectomy followed d by voriconazole - voriconazole was stopped due to photosensitive side effect. | Alive |
| Liu et al, 2021 (40) | N/D | 0.58 | F | China | Cough for 3 months, with fever and shortness of breath for 4 days. | N/D | X-ray revealed bilateral inflammation and local consolidation. CT revealed a large thick-walled cavity in the right lower lobe. | *Aspergillus* IgG was detected | N/D | CCPA | Oral voriconazole | Alive  Clinical and radiologic condition improved. |
| Zhang et al, 2021 (41) | N/D | 10 | F | China | Cough and haemoptysis | Recurrent pneumonia and allergic sinusitis | Aspergilloma | BAL culture negative  Histopathology confirmed CPAM  *Aspergillus* IgE and IgG positive | 2nd presentation possible ABPA | Simple Aspergilloma | Voriconazole for 8 weeks  After relapse – voriconazole, itraconazole and prednisone | Alive |
| Zhang et al, 2021 (41) | N/D | 13 | F | China | Cough and haemoptysis | Allergic sinusitis and skin allergy | Aspergilloma  nodules | Surgical pus culture positive for *A.* *fumigatus.*  Histopathology confirmed CPAM  *Aspergillus* IgE positive | Unremarkable | Simple aspergilloma and *Aspergillus*s nodule(s) | Voriconazole for 8 weeks.  After relapse  Voriconazole and Itraconazole for 6 months | Alive |
| Zhang et al, 2021 (41) | N/D | 9 | M | China | Cough and chest pain | Recurrent pneumonia, allergic sinusitis and skin allergy | Cavitary lesion | Histopathology confirmed CPAM  *Aspergillus* IgG positive | Unremarkable | CCPA | Voriconazole for 12 weeks | Alive |
| Zhang et al,  2021 (41) | N/D | 13 | M | China | Signs of pneumothorax | None | Pneumothorax | Histopathology confirmed *Aspergillus*  *Aspergillus* IgG positive | Unremarkable | N/D | Voriconazole for 8 weeks | Alive |
| Adeyemo et al, 2022 (42) | 2020 | 13 | F | Nigeria | Fever and cough | Pulmonary TB | cavitary lesions within both upper lung zones. | *A. flavus* was grown on culture | N/D | CCPA | Intravenous voriconazole | Died 23 days after admission |
| Ando et al, 2022 (43) | 2018 | 16 | M | Japan | Chest pain and shortness of breath | ABPA, Asthma | Chest Xray showed a cavity with consolidation in the right upper lung field. CT revealed a cavity with a fungal ball. 3 days later, more consolidation appeared around the cavity. | *A. fumigatus* was grown from the sputum and BALF culture. | Unremarkable | CCPA | Voriconazole with prednisone followed by the partial resection of the right upper lobe. | Alive |
| Davies et al, 2022 (44) | N/D | 9 | M | Nigeria | Recurrent cough, abdominal pain and fever. Chest pain, night sweats | None | multiple thick walled pulmonary cavitary nodules in both lungs | *Aspergillus* IgG was detected | N/D | CCPA | Oral itraconazole | Alive  Improvement within 48 hours |

# **Supplementary Table 2**: Other Possible CPA cases found in literature

| **Reference** | **Number of Cases** | **Additional Notes** |
| --- | --- | --- |
| Bulla 1968 (45) | Unknown | The paper mentions that it contains 16+ year olds, therefore it is possible that they have some cases of 17-year-olds with probable CPA, however they provide no radiological evidence, or any reason as to why they have done some of the procedures that they claim to have done. |
| Bogush, L 1973 (46) | Unknown | Several cases are described here, however there is insufficient details here to be able to tabulate any case from here. |
| Leophonte 1974 (47) | 1 - 12-year-old | This paper presents one 12-year-old, with a resection for an aspergilloma however does not provide enough detail about the case to tabulate for CPA |
| Aliazov 1985 (48) | Unknown | The paper mentions 41 patients between the ages of 15-60, 20 males and 21 females, therefore it is possible that some of them could have CPA |
| Eskenasy 1987 (49) | Possible 3 cases | The paper mentions 3 cases under the age of 20, therefore it is possible there are up to 3 CPA cases here, however they do not provide much information regarding it. |
| Coman 1987 (50) | 2 possible cases | The paper mentions 2 patients, one female and one male |
| Findik 2008 (51) | 1 | There is a lack of information regarding this, the paper mentions a patient child with an aspergilloma as a need for a thoracotomy, the main focus of the paper was to discuss the common conditions that require a thoracotomy |
| Ni 2008 (52) | Unknown | The paper mentions 40 cases of aspergillomas between the ages of 16-61 but the case series makes no clarification on the ages of any the cases. |
| Zhang 2009 (53) | 1 | There is a lack of information regarding this case. |
| Lieber 2015 (54) | 7 | The paper goes through experiences with thoroscopic surgeries in children, it mentions operating on 7 aspergilloma but provides no further details on these cases. |
| Elhattab 2021 (55) | 1 | This study talks about the use of thoracoscopic surgery for congenital lung malformation. One of the cases talks about an aspergilloma due to a persistent air leak within a child, however there is not enough information to tabulate this more in depth |

# **References**

1. Chaptal J, Negre E, Jean R, Rioux J, Pages A, Bonnet H, et al. [Apparently Primary Pulmonary Aspergilloma in a 4-Year-Old Girl]. Pediatrie (Bucur). 1963;18:759-70.

2. Miyamoto N, Kusumo H, Otsuka K, Mori S, Tsuneishi K, Hamazaki Y. [Experiences with the Treatment of 3 Cases of Lung Aspergillosis]. Kyoto Daigaku Kekkaku Kenkyusho Kiyo. 1963;12:1-10.

3. Krenmayr E. [Pulmonary Aspergillosis in Childhood]. Wien Med Wochenschr. 1964;114:473-4.

4. Besa G, Saccomani F. [Secondary pulmonary aspergilloma in a 4-year-old girl]. Minerva Pediatr. 1966;18(5):233-42.

5. Fouquet J, Cornu P, Cabrol C, Castelain G, Lerolle PF. [Intrabronchial aspergillosis. Complication of tuberculous primary infection]. Arch Fr Pediatr. 1966;23(1):37-45.

6. Rzepecki W, Rusin A, Sliwa Z, Zuk E. [Lung aspergilloma in a 11-year-old boy]. Gruzlica. 1967;35(9):905-8.

7. Isvanski M, Leposavic M, Simic P, Konecni R. [Pulmonary aspergillosis]. Srp Arh Celok Lek. 1970;98(11):1289-98.

8. Evans EG, Watson DA, Matthews NR. Pulmonary aspergillomata in a child treated with clotrimazole. Brit Med J. 1971;4(5787):599-600.

9. Berger I, Phillips WL, Shenker IR. Pulmonary aspergillosis in childhood. A case report and discussion. Clin Pediatr (Phila). 1972;11(3):178-82.

10. Beauvais P, Binet JP, Neel P, Brissaud HE. [Bronchopulmonary aspergilloma in children and bronchogenic cyst. A new case]. SEM HOP. 1975;-51(46):2775-9.

11. Kane PA, Sarr AM, Courbil JL, Ducloux M, Derrien JP, Coly D, et al. [Pleuro-pulmonary aspergillosis in African blacks (apropos of 5 cases observed in Dakar)]. Bull Soc Med Afr Noire Lang Fr. 1976;21(3):363-75.

12. Rzepecki W. [Surgery in cases of unusual forms of pulmonary aspergillosis]. Pol Przegl Chir. 1977;49(11):1113-20.

13. Bundgaard A, Schonheyder H, Bech-Jansen P. [Pulmonary aspergillosis with abscess formation]. Ugeskr Laeger. 1983;145(26):2029-30.

14. Grignet JP, Pagnier F, Faillon JM, Leduc M, Cecile JP, Bauduin JL, et al. [A case of pulmonary sequestration associated with Aspergillus pathology]. LARC Med. 1983;3(10):689-90, 93-5.

15. David A, Melon V, Cohen JY. [Iconographic rubric. Pulmonary aspergilloma]. Arch Fr Pediatr. 1986;43(8):641-2.

16. Castaneda-Ramos SA, Aguilar-Benavides S, Ramos-Solano F, Arenas-Arechiga P, Garzon-de la Mora MA. [Pulmonary aspergillosis]. Bol Med Hosp Infant Mex. 1989;46(1):51-5.

17. Lui RC, Inculet RI. Job's syndrome: a rare cause of recurrent lung abscess in childhood. Ann Thorac Surg. 1990;50(6):992-4.

18. Hiura K, Katoh O, Kawashima M, Nakata H, Aoki Y, Nakahara Y, et al. [A case of spontaneous pneumothorax due to rupture of bleb infected with aspergillosis]. Nihon Kyobu Shikkan Gakkai Zasshi. 1993;31(3):364-7.

19. Karim M, Alam M, Shah AA, Ahmed R, Sheikh H. Chronic invasive aspergillosis in apparently immunocompetent hosts. Clin Infect Dis. 1997;24(4):723-33.

20. Santambrogio L, Nosotti M, Pavoni G, Harte M, Pietogrande MC. Pneumatocele complicated by fungal lung abscess in Job's syndrome. Successful lobectomy with the aid of videothoracoscopy. Scand Cardiovasc J. 1997;31(3):177-9.

21. Wolach B, Eliakim A, Gottesman G, Yellin A. Pulmonary aspergillosis in a child with hyperimmunoglobulin E syndrome. Clin Infect Dis. 1998;26(1):204-5.

22. Kul'ko AB, Dubrovskii AV, Kuz'min DE. [A case of pulmonary aspergillosis caused by Aspergillus terreus]. Probl. 2003(12):30-2.

23. Zhao SY, Jiang ZF, Xu SY. Diagnosis and treatment of chronic pulmonary aspergillosis in 4 children. [Chinese]. Zhonghua er ke za zhi. 2005;Chinese journal of pediatrics. 43(2):113-7.

24. Rubilar O L, Maggiolo M J, González V R, Girardi B G, Mendoza N C, Vildósola S C. Chronic pulmonary aspergillosis associated to multiple airway papillomatosis: Case-report and literature review. Revista Chilena de Pediatria. 2006;77(4):382-7.

25. Arai H, Rino Y, Fujii K, Yamada T, Suganuma N, Yukawa N, et al. [Surgical treatment for pulmonary aspergillosis with hyper immunoglobulin-E syndrome; report of a case]. Kyobu Geka. 2007;60(12):1122-5.

26. Chtourou I, Bahri Zouari I, Gouiaa N, Fakhfakh I, Charfi S, Hadj Kacem A, et al. Oxalose et aspergillose pulmonaires nécrosantes : à propos de deux observations. Journal de Mycologie Médicale. 2007;17(2):122-5.

27. Kuruvilla S, Saldanha R, Joseph LD. Recurrent respiratory papillomatosis complicated by aspergillosis: a case report with review of literature. J Postgrad Med. 2008;54(1):32-4.

28. Yonker LM, Mark EJ, Canapari CA. Aspergilloma in a patient with an occult congenital pulmonary airway malformation. Pediatr Pulmonol. 2012;47(3):308-10.

29. Pan JB, Hou YH, Yin PZ. A case report of hydatid cysts containing aspergillus. J Thorac Dis. 2013;5(2):E25-7.

30. Kane S, Pinto JM, Dadzie CK, Dawis MA. Aspergilloma caused by Aspergillus versicolor. Pediatr Infect Dis J. 2014;33(8):891.

31. Stojnic N, Vasiljevic ZV, Djuricic SM, Arsenijevic VA, Minic P. Chronic necrotizing pulmonary aspergillosis in an immunocompetent, obese 10-year-old boy. Turk J Pediatr. 2014;56(6):654-7.

32. Emiralioglu N, Ozcan HN, Orhan D, Haliloglu M, Cengiz AB, Yalcin EE, et al. Chronic necrotizing pulmonary aspergillosis in an immunocompetent patient after the surgery of hydatid cyst. Tuberk Toraks. 2017;65(2):157-60.

33. Chandrakar J, Agrawal A, Pandey V, Das A, Bhagat K. Hydatid cyst with superadded aspergillosis in lung: A rare case report. Indian Journal of Pathology and Microbiology. 2018;61(5):S67.

34. Isnard M, Hullo E, Robert Y, Piolat C, Durand C, Lantuejoul S, et al. [Post-traumatic pulmonary aspergilloma]. Rev Mal Respir. 2018;35(3):342-6.

35. McDowell MD, Estrellado-Cruz WL. Chronic cavitary pulmonary aspergillosus with aspergilloma in a previously healthy teenager. American Journal of Respiratory and Critical Care Medicine Conference: American Thoracic Society International Conference, ATS. 2018;197(MeetingAbstracts).

36. Yu Y, Esther CR. Invasive cavitary aspergillus infection complicated by abpa. American Journal of Respiratory and Critical Care Medicine Conference. 2019;199(9).

37. Aboksari MS, Safavi M. Concomitant Pulmonary Cystic Echinococcosis and Aspergillosis in a Male Child. J Trop Pediatr. 2020;66(6):645-7.

38. Rana M, Khan S, Pervez M, Fatimi S. Giant Pneumatocele Secondary to Aspergillus Nidulans in Autosomal Dominant Hyper-Ige Syndrome Child. Chest. 2020;157(6):A33.

39. Abo YN, Gwee A, Osowicki J. Rare Infant Case of Pulmonary Aspergilloma Highlighting Common Challenges With Voriconazole Dosing. Pediatr Infect Dis J. 2021;40(3):227-30.

40. Liu Z, Qiao L, Li D, Yu L, Yi L. Chronic cavitary pulmonary aspergillosis in an infant. Lancet Infect Dis. 2021;21(12):1757.

41. Zhang XY, Zhou CJ, Tang XL, Liu H, Zhao SY, Chen CH. [Analysis of four cases with congenital pulmonary airway malformation complicated by chronic pulmonary aspergillosis]. Zhonghua Er Ke Za Zhi. 2021;59(10):871-5.

42. Adeyemo AT, Obadare TO, Edward SS, Ibrahim AO, Irek EO, Amupitan AA, et al. Clinical neglect of aspergillosis in pulmonary tuberculosis coinfection: a case report of avoidable mortality in a resourceconstrained setting. African Journal of Clinical and Experimental Microbiology. 2022;23(3):323-9.

43. Ando E, Nakasuka T, Kubo T, Taniguchi A, Ninomiya K, Kato Y, et al. Pulmonary Aspergilloma and Allergic Bronchopulmonary Aspergillosis Following the 2018 Heavy Rain Event in Western Japan. Intern Med. 2022;61(3):379-83.

44. Davies AA, Adegbite IA, Akintan PE, Ibrahim UO, Adekoya AO, Oladele RO. Chronic cavitary pulmonary aspergillosis in an immunocompetent child. Med Mycol Case Rep. 2022;37:33-6.

45. Bulla A, Kercea V, Gologan I. [Occurrence of secondary bronchopulmonary mycosis manifestations in patients with treated tuberculosis]. Z Tuberk Erkr Thoraxorg. 1968;128(1):254-6.

46. Bogush LK, Strel'tsov VP. [Diagnosis and surgical treatment of fungal lung diseases]. Klin Med (Mosk). 1973;51(7):84-8.

47. Leophonte P, Pribat JP, Carles P, Gaillard J, Eschapasse H. [Pulmonary aspergillomas. Diagnostic, prognostic and therapeutic problems. (Apropos of 60 cases observed in a surgical department)]. Cah Med. 1974;15(5):253-9.

48. Aliazov SI, Gurbanaliev IG, Manafov SS. [Surgical treatment of bronchopulmonary aspergillosis]. Grudn Khir. 1985(2):17-9.

49. Eskenasy A, Molan M. Aspergillary mycetomas ("aspergillomas") of the lung. Histopathologic and mycologic investigations in 45 cases. Morphol Embryol (Bucur). 1987;33(3):193-203.

50. Coman C, Stan A, Micu V, Dimitriu IM, Coman B. [Treatment of pulmonary aspergilloma]. Rev Chir Oncol Radiol O R L Oftalmol Stomatol Chir. 1987;36(5):363-71.

51. Findik G, Gezer S, Sirmali M, Turut H, Aydogdu K, Tastepe I, et al. Thoracotomies in children. Pediatr Surg Int. 2008;24(6):721-5.

52. Ni ZY, Xu J, Zhou M, Cao F. Clinical analysis of 40 cases of pulmonary aspergilloma. [Chinese]. Zhonghua jie he he hu xi za zhi = Zhonghua jiehe he huxi zazhi = Chinese journal of tuberculosis and respiratory diseases. 2008;31(9):675-7.

53. Zhang XY, Zhao SY, Qian SY, Hu YH, Zeng JJ, Jiang ZF. [Diagnosis and treatment of invasive pulmonary aspergillosis in 21 children with non-hematologic diseases]. [Chinese]. Zhonghua er ke za zhi. 2009;Chinese journal of pediatrics. 47(10):730-4.

54. Lieber J, Urla CI, Baden W, Schafer J, Kirschner HJ, Fuchs J. Experiences and challenges of thorcoscopic lung surgery in the pediatric age group. Int J Surg. 2015;23(Pt A):169-75.

55. Elhattab A, Elsaied A, Wafa T, Jugie M, Delacourt C, Sarnacki S, et al. Thoracoscopic surgery for congenital lung malformations: Does previous infection really matter? J Pediatr Surg. 2021;56(11):1982-7.
